# N- and O- Doped Porous Carbon Nanosheets Prepared from Templating Methodology for Supercapacitors

**DOI:** 10.3390/polym17091198

**Published:** 2025-04-27

**Authors:** Baoning Zhu, Jinghua Liu, Qijun Zhong, Yaru Wen, Qianqian Dong, Yuhao Li, Qianqian Jin, Yao Lu

**Affiliations:** 1 Liuzhou Key Laboratory of New Energy Vehicle Power Lithium Battery, Guangxi Engineering Research Center for Characteristic Metallic Powder Materials, School of Electronic Engineering, Guangxi University of Science and Technology, Liuzhou 545000, China; 18505526400@163.com (B.Z.); zqj17878906682@163.com (Q.Z.); 13906438619@163.com (Y.W.); isdongqq@163.com (Q.D.); q2476166290@126.com (Y.L.); qqjin10s@139.com (Q.J.); 2 Guangxi Key Laboratory of Green Processing of Sugar Resources, College of Biological and Chemical Engineering, Guangxi University of Science and Technology, Liuzhou 545006, China

**Keywords:** sheet structure, porous carbon, nitrogen–oxygen doping, supercapacitor

## Abstract

Heteroatom-doped biomass-derived porous carbon materials show promising applications as electrode components in energy storage technologies. In this investigation, we present a template-assisted pyrolysis procedure to fabricate nitrogen–oxygen dual-doped carbon materials. Firstly, the precursor and template initially polymerized to form a white jelly-like gel, which was freeze-dried to create a nanosheet-assembled structure. Subsequent high-temperature pyrolysis induced the formation of a porous structure with nanosheet morphology. The CMC-ZnK sample derived from the dual template of potassium citrate and zinc acetate pyrolyzed at 800 °C exhibits optimal electrochemical performance, delivering a specific capacitance of 271.4 F g^−1^ at 1 A g^−1^ in a three-electrode configuration, along with outstanding rate capability (90% retention, 244 F g^−1^ at 10 A g^−1^). The constructed supercapacitor demonstrated an energy density of 6.5 Wh kg^−1^ under a power density of 500 W kg^−1^. Furthermore, this study systematically investigated the performance variation mechanisms at different temperatures, revealing the relationship between structural composition and temperature in biomass materials.

## 1. Introduction

At present, electrical energy stores play an important role for human beings, and there are various ways to store electrical energy, such as chemical batteries, solar cells, and supercapacitors [1]. Among them, supercapacitors offer greater transient response, exceptional power density, minimal weight, compact dimensions, and reduced internal resistance, making them well suited for a broad spectrum of uses [2]. Supercapacitors are generally classified into three primary categories based on their energy storage principles: electrical double-layer capacitors (EDLCs), pseudocapacitors (PCs), and hybrid battery supercapacitors (HBSs) [3]. EDLCs predominantly utilize carbon-based materials, including carbon nanotubes, activated carbon, and graphite, as their electrode components. During the charge and discharge processes, EDLCs operate through a mechanism that does not involve charge transfer between the electrolyte and the electrode. Instead, they store electrical charge via a non-Faradaic process, which is characterized by the physical adsorption and desorption of ions at the electrode–electrolyte interface. In contrast, PCs exhibit a distinct energy storage mechanism that involves rapid and reversible Faradaic reactions, akin to the electrochemical processes observed in batteries. These reactions entail the transfer of charge across the electrode–electrolyte boundary, resulting in an alteration of the electrode material’s oxidation state. HBSs integrate the advantageous features of both EDLCs and PCs, integrating the superior power density and extended cycle stability of EDLCs with the increased energy density offered by the Faradaic mechanisms of PCs. The electrode materials exert a profound influence on the performance enhancement of supercapacitors. Porous carbon is currently a crucial electrode material, among which biomass-derived porous carbon has attracted extensive research attention due to its abundant sources, relatively high specific capacitance, and excellent cycling stability [4]. Biomass-derived porous carbon optimization of their properties is governed by multiple critical factors including dopant selection, concentration control, and specific doping ratios [5]. Particularly, heteroatom doping with elements such as nitrogen, phosphorus, oxygen, and boron has proven effective in significantly enhancing capacitive performance through electronic structure modification [6]. Advanced doping techniques involving diatomic or polyatomic elements further improve material characteristics by creating synergistic effects between different heteroatoms [7]. These dopant atoms preferentially substitute carbon atoms within the lattice structure, thereby modifying electron transfer dynamics and surface chemistry to enhance electrochemical behavior. For instance, Zhou et al. fabricated porous carbon electrode materials from lignin through the co-doping of nitrogen and sulfur during carbonization and activation. A maximum specific capacitance of 391.4 F g^−1^ was attained at a current density of 0.5 A g^−1^, with a cycling stability retention of 94.2% over 20,000 cycles [8]. Nie and co-workers successfully prepared biomass-derived porous carbon (N_2_PC_5_) using corn stover as the carbon source and ZnCl_2_ as the activating agent. The resulting material exhibited a specific capacitance of 321.5 F g^−1^ at a current density of 0.5 A g^−1^. Notably, the supercapacitor demonstrated excellent cycling stability with 93% capacitance retention after 10,000 charge–discharge cycles [9].

Chitosan, the deacetylated derivative of chitin from crustacean exoskeletons, has attracted significant research interest in developing various electrolyte/electrode materials for supercapacitors due to its biodegradability, abundant amino functional groups, environmental friendliness, and low extraction costs [10]. A variety of chitosan-based electrodes have been created. It is important to modify the permeable framework of chitosan-based carbon to promote its supercapacitive properties [11]. Heteroatom doping represents an effective strategy for optimizing both the electrochemical performance and pore structure of polysaccharide-derived carbon materials. An alternative fabrication strategy utilizes templating techniques to create porous structures. After removing the template, this method can leave an interconnected carbon skeleton, further enriching the porous structure. Through the template strategy, the obtained porous structure can favor electrolyte penetration and exposure of electrochemical active sites. For example, Zhu et al. [12] fabricated nitrogen-rich porous carbonaceous materials with a specific capacitance of 305 F g^−1^ at a current density of 0.5 A g^−1^. Minakshi et al. significantly enhanced the performance of CoMoO_4_ by incorporating chitosan crosslinked with glutaraldehyde. Compared to the chitosan-free counterpart, the modified material exhibited a 4-fold increase in capacitance (from 17 F g^−1^ to 81 F g^−1^) [13]. Luo et al. developed porous carbon derived from chitosan hydrogel via a rapid one-step method for high-rate supercapacitors [14]. Among their materials, CHPC-7 exhibited the best performance due to its optimized pore structure. To our knowledge, there is scarce research on the synthesis of chitosan-derived porous carbon utilizing a dual-template approach involving potassium citrate and zinc acetate.

Herein, we report an innovative heteroatom doping strategy utilizing a dual-template synergistic approach to fabricate nitrogen/oxygen co-doped porous carbon materials [15]. The approach employs sustainable chitosan and melamine as dual N/C precursors, combined with potassium citrate and zinc acetate to construct a dual-template system. The synthesis involves a crosslinking reaction between chitosan and glutaraldehyde, while incorporating melamine as an additional nitrogen source along with potassium citrate and zinc acetate as structural templates. Notably, both chitosan and melamine molecules contain amino functional groups, whereas glutaraldehyde possesses dialdehyde moieties. These molecular components undergo crosslinking reactions between amino and hydroxyl groups, forming an interpenetrating network structure. Subsequent carbonization at optimized temperatures successfully constructs a porous carbon architecture. This innovative methodology yields N/O co-doped porous materials with significantly enhanced electrochemical performance.

## 2. Materials and Methods

### 2.1. Manufacturing of Porous Carbon

The required experimental reagents have deionized water. Acetic acid was obtained from Sichuan Xilong Science Co., Ltd. (Chengdu, China), at analytical grade. Chitosan, Glutaraldehyde, potassium citrate, and zinc acetate were procured from Shanghai Macklin Biochemical Co., Ltd. (Shanghai, China), at analytical grade. Melamine was supplied by Shanghai Aladdin Biochemical Technology Co., Ltd. (Shanghai, China), at analytical grade. First, 200 μL of acetic acid was added to 20 mL of deionized water and it was mixed well. A 0.1 g sample of chitosan was dissolved in 20 mL of a 2 vol% acetic acid solution. The solution was stirred until it was transparent and clear. Then, 0.3 g of melamine was added until it was completely dissolved. The solution was mixed completely and stirred well, and 0.2 g of potassium citrate and 0.1 g of zinc acetate were added in turn. Finally, glutaraldehyde was added drop by drop and stirred well to form hydrogel. Carbonization: All samples were pre-frozen in a −20 °C freezer for 12 h. Subsequently, the hydrogel underwent freeze-drying at −50 °C for 24 h. The freeze-dried aerogels were carbonized at different temperatures under the protective atmosphere of nitrogen. Finally, the required porous carbon material was obtained. The chitosan–melamine porous carbon synthesized at 800 °C without any activating agents is denoted as CMC. The material using a dual template (potassium citrate and zinc acetate) is labeled as CMC-ZnK, while those prepared with a single template (either potassium citrate or zinc acetate alone) are designated as CMC-K and CMC-Zn, respectively. The CMC-ZnK samples prepared at pyrolysis temperatures of 700 °C, 800 °C, and 900 °C are designated as CMC-ZnK-x, where x represents the pyrolysis temperature.

### 2.2. Physical Characterization

The structural characteristics of the synthesized materials were systematically investigated using a suite of advanced analytical methods. An Ultima IV diffractometer (RIGAKU, Tokyo, Japan) with Cu Kα radiation was employed for X-ray diffraction (XRD) analysis. Brunauer–Emmett–Teller (BET) surface area measurements and pore size distribution analysis were conducted using a Micromeritics ASAP 2460 instrument (Micromeritics Instrument Co., Ltd., Shanghai, China) through nitrogen adsorption–desorption isotherms at 77 K. Morphological features and surface structures were examined using field emission scanning electron microscopy (FE-SEM, Gemini 300, Zeiss, Jena, Germany) operating at 5 kV. High-resolution transmission electron microscopy (HR-TEM) observations were carried out on a Titan ETEM G2 system (FEI, Hillsboro, OR, USA) with an accelerating electric potential range of 80–300 kV, enabling detailed microstructural analysis at atomic resolution. Chemical composition and surface electronic states were characterized by X-ray photoelectron spectroscopy (XPS) using an A Scientific K-Alpha+ spectrometer (Thermo Fisher Scientific, Waltham, MA, USA) equipped with monochromatic Al Kα radiation. Furthermore, Raman spectroscopic analysis was performed with a RST2-301-SMS Raman spectroscopy system (RST2-301-SMS, Zhuolihanguang, Beijing, China) equipped with a 532 nm laser excitation source to identify characteristic vibrational modes and chemical bonding configurations.

### 2.3. Electrochemical Analysis

The electrochemical behavior was analyzed using an Ivium electrochemical analyzer. Electrochemical testing includes CV (cyclic voltammetry), GCD (galvanostatic charge/discharge), and EIS (electrochemical impedance spectroscopy). The electrochemical cell consists of a 6 M potassium hydroxide electrolyte within a three-electrode configuration. The counter electrode is platinum foil and Hg/HgO is a reference electrode. The working electrode is supported by nickel foam coated with an active substance. Activated acetylene black, active substance, and PTFE were mixed in ethanol solvent in a composition with an 8:1:1 mass distribution. The mixture was then applied onto a nickel foam substrate. The carrier was dehydrated in a 100 °C drying oven for 10 h and then pressurized at 10 MPa for 30 s to make a functional electrode. The CV scans were conducted within a potential window of −1.0 to 0 V. The electrochemical impedance spectrum frequency range was set to 0.01~10 kHz [16]. As per the formula Cs = (I × ∆t)/(m × ∆V), the capacitance per unit mass of the active substance (Cs, F g^−1^) was analyzed from the GCD curve, in which I (A) corresponds to the discharge current, ∆t (s) indicates the discharge time, m (g) refers to the active material’s mass, and ∆V (V) represents the voltage shift during the discharge phase. At last, a symmetric supercapacitor was successfully assembled using the CMC-ZnK-800 material. The energy density (E_-cell_, Wh kg^−1^) and power density (P_-cell_, W kg^−1^) of the capacitor were calculated using the equations E_-cell_ (Wh kg^−1^) = (C_-cell_ × V^2^)/(2 × 3.6) and P_-cell_ (W kg^−1^) = E_-cell_ × 3600/Δt, respectively, where C_-cell_ is the specific capacitance of the capacitor, V is the cell operating potential (V), and Δt is the discharge time (s) [17].

**Figure 1:**
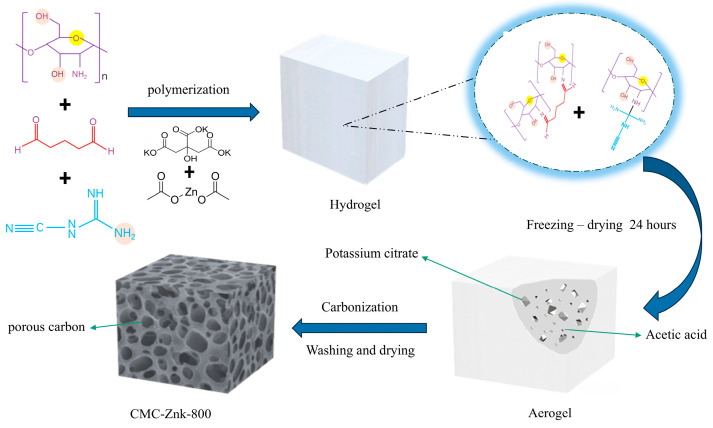
Preparation process of CMC-ZnK-800 porous carbon material.

The underlying polymerization mechanism involves the crosslinking of chitosan and melamine mediated by glutaraldehyde, resulting in the formation of a milky-white, jelly-like hydrogel as shown in Figure 1. Potassium citrate and acetic acid are uniformly distributed in the gel. The amino groups of chitosan undergo an amination reaction with the aldehyde functionalities of glutaraldehyde during the aggregation process, and this structural modification serves to enhance structural stability [18]. Moreover, the decomposition reaction of potassium citrate at high temperatures produces potassium carbonate, carbon dioxide, and water [19,20]. Further high-temperature decomposition of potassium carbonate produces potassium oxide and carbon dioxide (1). During the high-temperature process, the generated K can be intercalated into the carbon skeleton, thereby expanding the carbon microstructure (2) [21]. Subsequently, the residual potassium oxide remains in the material as a hard template. Similar to the reaction with potassium citrate, at elevated temperatures, zinc acetate breaks down, producing gaseous substances and resulting in the formation of zinc oxide (3). Zinc oxide partially evaporates into Zn vapor, while the remaining particles may serve as templates, further contributing to pore formation [22]. After carbonization and activation modification, a porous carbon structure is formed.

(1) $$K_{2}{\mathrm{CO}}_{3}\;\overset{△}{\to }K_{2}O\;+{\mathrm{CO}}_{2}↑$$

(2) $$K_{2}O\;+\;2C\;\overset{△}{\to }2K\;+\;\mathrm{CO}↑$$

(3) $$\mathrm{Zn}({\mathrm{CH}}_{3}\mathrm{COO}{)}_{2}\;\overset{△}{\to }\mathrm{ZnO}\;+\;({\mathrm{CH}}_{3}\mathrm{CO}{)}_{2}O$$

## 3. Results

**Figure 2:**
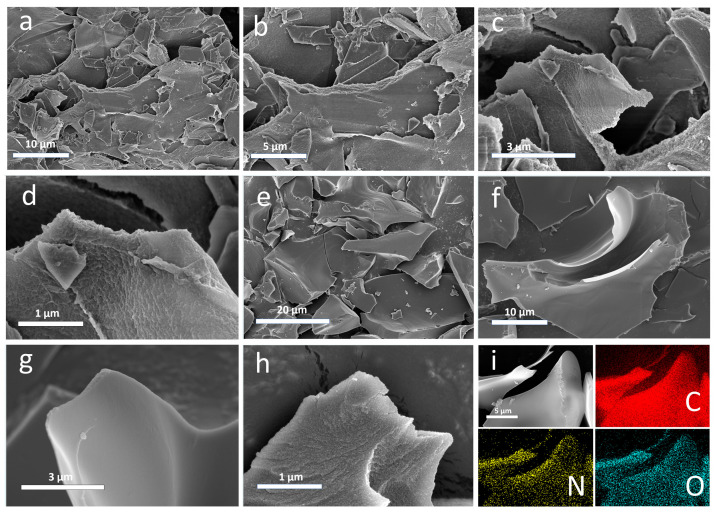
(**a**–**d**) SEM image of CMC-ZnK-800 before high temperature carbonization; (**e**–**h**) SEM image of CMC-ZnK-800 after high temperature carbonization; (**i**) elemental mapping of C, N, and O.

A more detailed investigation of the surface morphology of CMC-ZnK-800 material before and after the carbonization process at high temperatures was performed via TEM. As displayed in Figure 2a–d, the microstructure of the CMC-Znk-800 material before high-temperature carbonization exhibits a rough lamellar stacking structure. There is evidence that chitosan, melamine, and glutaraldehyde successfully cross-polymerize with each other. Porous structures rich in porosity are observed in rough sheet stacks. After high-temperature carbonization protected by nitrogen at 800 °C, it can be observed that the rough layered stacking structure before carbonization is well preserved, as displayed in Figure 2e–h. The layered architectures with substantial surface area facilitate the adsorption of electroactive ions and optimize interfacial contact with the electrolyte, thereby enhancing the electrochemical performance of carbon-based electrodes [23,24]. Additionally, the layered structure can effectively shorten the transmission distance between ions, and the porous structure can greatly increase the expanded surface area of the material supports optimal ion conduction [25].

**Figure 3:**
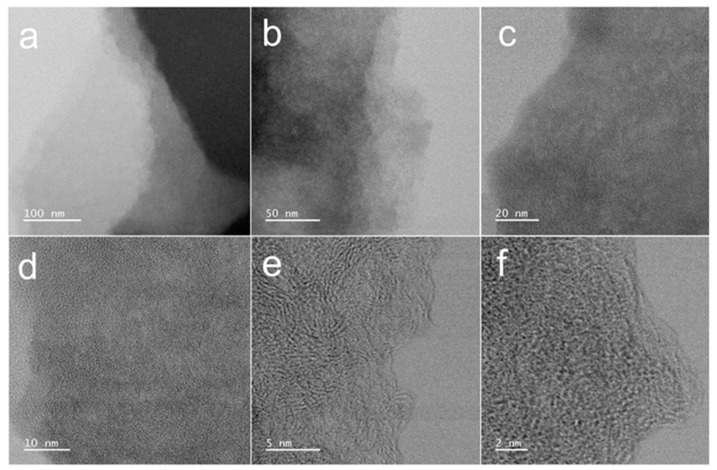
(**a**–**c**) TEM images of CMC-ZnK-800 at different magnifications; (**d**–**f**) HR-TEM images of CMC-ZnK-800 at different magnifications.

As revealed by TEM analysis (Figure 3), the material surface exhibits a distinctive nanostructured morphology characterized by abundant nanoporous architectures resembling water-wave patterns. The surface topography demonstrates significant roughness, with numerous fine particles uniformly distributed across the material surface. Furthermore, the material exhibits a well-defined hierarchical structure consisting of stacked nanolamellar sheets. These structural features collectively indicate the presence of extensive microporous networks within the CMC-ZnK-800 composite, which is consistent with the morphological characteristics previously observed through SEM analysis.

**Figure 4:**
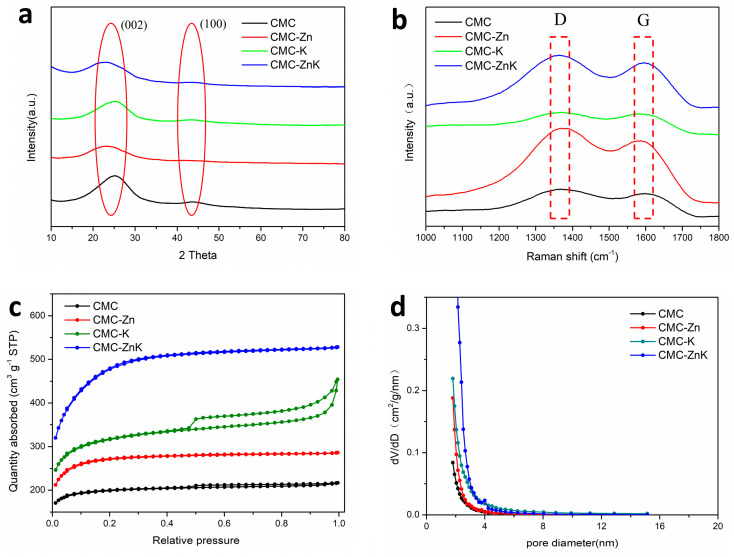
(**a**) XRD patterns of CMC, CMC-Zn, CMC-K, and CMC-ZnK; (**b**) Raman spectra of CMC, CMC-Zn, CMC-K, and CMC-ZnK; (**c**) N_2_ adsorption/desorption isotherms of CMC, CMC-Zn, CMC-K, and CMC-ZnK; (**d**) PSD curves of CMC, CMC-Zn, CMC-K, and CMC-ZnK.

Figure 4a presents the XRD patterns of CMC, CMC-Zn, CMC-K, and CMC-ZnK. All four materials exhibit a strong diffraction peak at approximately 23° and a weaker peak around 44°, corresponding to the (002) and (100) crystallographic planes of graphitic carbon, respectively. The (002) diffraction peak observed in the XRD pattern confirms the presence of locally graphitized microcrystals, while the overall carbon matrix remains predominantly amorphous with low crystallinity [26]. These features confirm the partial retention of graphitic ordering within the carbon matrix. To further quantify the graphitic characteristics, Raman spectroscopy was employed (Figure 4b). Distinct D and G bands were observed at ~1350 cm^−1^ and ~1600 cm^−1^, respectively. The D band arises from structural defects and disordered carbon domains, while the G band corresponds to the in-plane vibrational mode of sp^2^-hybridized carbon atoms, serving as an indicator of graphitic ordering. The intensity ratio (I_D_/I_G_) was calculated to evaluate the relative graphitization degree of the materials. The measured ratios for the samples were as follows: 1.16 for pristine CMC, 1.29 for Zn-activated CMC-Zn, 1.10 for K-activated CMC-K, and 1.09 for dual-activated CMC-ZnK. These results reveal that zinc acetate introduction increases structural disorder, likely due to lattice distortion induced by Zn^2+^ incorporation. Conversely, potassium citrate addition enhances graphitization, potentially through catalytic graphitization mechanisms during pyrolysis. Notably, the synergistic combination of both precursors achieves the lowest I_D_/I_G_ ratio, indicating optimized graphitic ordering. This enhanced crystallinity is critical for improving electrical conductivity, a key determinant of electrochemical performance in carbon-based materials [27]. Nitrogen adsorption–desorption measurements were conducted on the four materials to investigate the influence of mixed pore-forming agents on the porosity and hierarchical structure of carbon-based materials for supercapacitor applications. As shown in Figure 4c, all four isotherms exhibit combined Type I and Type IV characteristics. The appearance of slight hysteresis loops indicates the coexistence of microporous and mesoporous structures in these materials. The specific surface areas of CMC, CMC-Zn, CMC-K, and CMC-ZnK were determined to be 203.4 m^2^ g^−1^, 275.3 m^2^ g^−1^, 346.0 m^2^ g^−1^, and 507.8 m^2^ g^−1^, respectively. The undoped CMC sample displayed the flattest isotherm, corresponding to the lowest specific surface area among the four materials. The CMC-ZnK composite exhibited a typical Type IV isotherm, with a rapid rise in the medium-pressure region, confirming the presence of mesopores [28,29]. The mesoporous structure facilitates efficient ion transport and diffusion, contributing to superior rate capability and high power density, suggesting that potassium citrate primarily induces mesopore formation. The pore size distribution of CMC-ZnK (Figure 4d) was predominantly in the 2–4 nm range, while micropores remained the dominant pore structure. Micropores enhance charge storage capacity, enabling high specific capacitance, whereas macropores effectively reduce mass transport resistance within the carbon framework [30]. The dual doping strategy significantly optimized the microporous architecture, resulting in the highest surface area per unit mass among the four samples. Furthermore, the mesoporous structure not only improves electrolyte ion storage but also provides efficient ion transport pathways, substantially reducing ion/electron diffusion distances [31,32]. These synergistic effects collectively contributed to the enhanced electrochemical performance of the material. 

**Figure 5:**
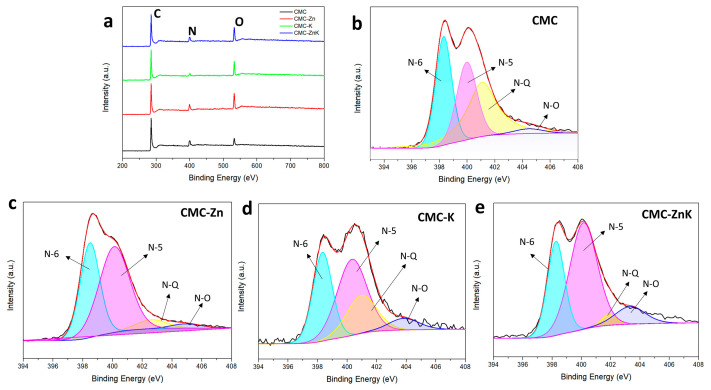
(**a**) XPS spectra of CMC, CMC-Zn, CMC-K, and CMC-ZnK; (**c**) N1s spectra of (**b**) CMC, (**c**) CMC-Zn, (**d**) CMC-K, and (**e**) CMC-ZnK.

The chemical compositions and surface element contents of CMC, CMC-Zn, CMC-K, and CMC-ZnK were investigated by XPS. Figure 5a shows typical XPS spectra of the four materials. All materials are made up of three elements, carbon, hydrogen and oxygen. The corresponding carbon, nitrogen, and oxygen contents are shown in Table 1. All doped materials exhibit a gradual consumption of nitrogen content and an increase in oxygen content during the carbonization process. The results show the addition of potassium citrate can rapidly reduce the nitrogen content of the materials. Compared to potassium citrate, zinc acetate can more gently reduce the nitrogen content of the material while retaining a higher proportion of nitrogen. The nitrogen content in CMC-ZnK decreased slightly, and the oxygen content increased. However, even retaining a small amount of heteroatoms (N) can have a positive impact on the enhancement of electrochemical performance [33]. Nitrogen atoms contribute to pseudo capacitance through redox reactions and enhance the wettability of the active electrode material, thus improving electrochemical performance. CMC-ZnK demonstrates the optimal electrochemical performance, which can be attributed to the high oxygen content in CMC-Zn and the near-optimal nitrogen-to-oxygen (N/O) ratio in CMC-ZnK, thereby providing abundant active sites. Figure 5b–e reveal four distinct bonding configurations: pyridine nitrogen (N-6, 398.5 eV), pyrrole nitrogen (N-5, 400.2 eV), graphitic nitrogen (N-Q, 401.0 eV), and oxidized nitrogen (N-O, 403.0 eV). Notably, the N-5 and N-6 configurations contribute significantly to the formation of structural defects and electrochemically active sites, which synergistically enhance pseudocapacitance through redox reactions. The comparative analysis reveals that the N-Q content persistently decreases regardless of the activating agent source (zinc acetate or potassium citrate). In contrast, the proportion of N-5 species exhibits a marked increase under both doping conditions, while the N-6 content remains relatively stable. This systematic variation indicates that both zinc acetate and potassium citrate preferentially promote the formation of pyrrolic nitrogen configurations. The dual doping strategy employing both zinc acetate and potassium citrate synergistically amplifies the N-5 content, creating abundant structural defects and active sites that substantially improve the material’s electrochemical performance.

**Figure 6:**
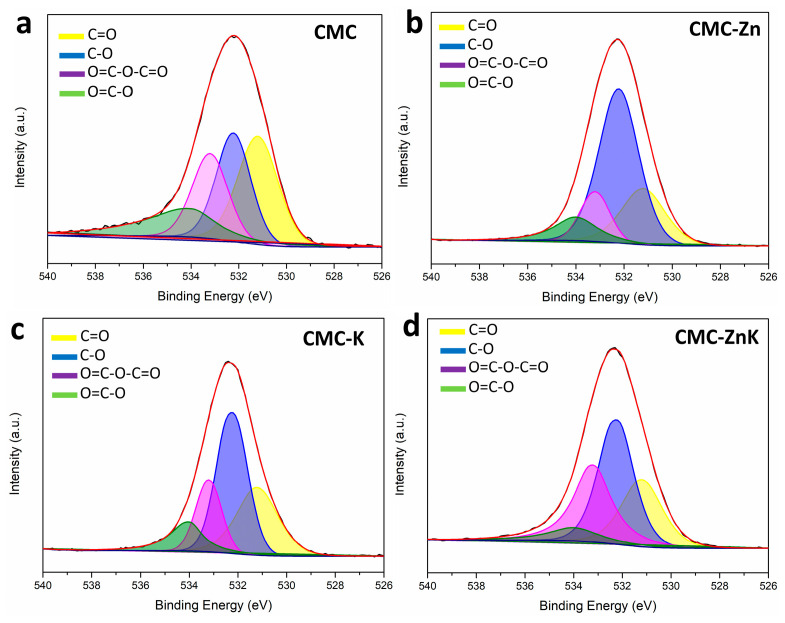
O1s spectra of (**a**) CMC, (**b**) CMC-Zn, (**c**) CMC-K, and (**d**) CMC-ZnK.

As shown in Figure 6, the high-resolution O 1s spectra of the CMC-ZnK material reveal four distinct peaks, corresponding to different oxygen-containing functional groups. These peaks can be deconvoluted into four components, representing C=O (531.2 eV), C-O (532.1 eV), O=C-O-C=O (533.2 eV), and O=C-O (534.0 eV), respectively. Functional groups with oxygen can boost the specific capacitance of carbon materials by contributing to Faradaic pseudocapacitance [34]. However, excessive oxygen content may block the pores of the material, leading to diminished electrochemical performance [35]. Therefore, the appropriate oxygen content is critical for achieving improved properties of CMC-ZnK materials.

**Figure 7:**
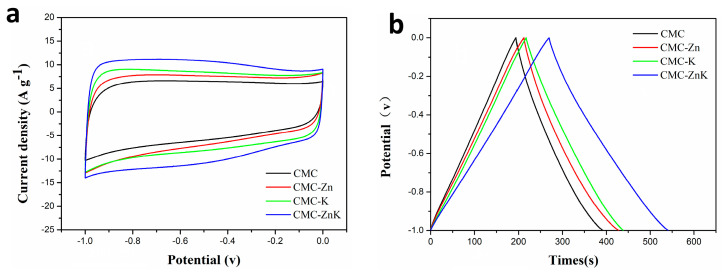
(**a**) CV curves of CMC, CMC-Zn, CMC-K, and CMC-ZnK electrodes tested at constant scan rate of 50 mV∙s^−1^; (**b**) GCD curves of CMC, CMC-Zn, CMC-K, and CMC-ZnK electrodes measured at constant current density of 1 A g^−1^.

The electrochemical performance of CMC, CMC-K, CMC-Zn, and CMC-ZnK materials was systematically evaluated using a three-electrode configuration with a 6 M KOH electrolyte. As shown in Figure 7a, all CV curves exhibit quasi-rectangular shapes, indicating typical electric double-layer capacitor behavior. GCD profiles in Figure 7b demonstrate the discharge duration, which is directly proportional to specific capacitance. The specific capacitance of CMC-ZnK materials significantly improves after simultaneously doping with potassium citrate- or zinc acetate-doped materials. The optimal electrochemical performance corresponds to the material exhibiting the largest specific surface area among the four samples, demonstrating a strong correlation between electrochemical behavior and BET-derived surface characteristics. Rate capability tests (Figure S1) were conducted to evaluate the specific capacitances of four materials at current densities ranging from 1 to 10 A g^−1^. At 1 A g^−1^. The calculated specific capacitances were calculated as 198.2, 217.2, 221.8, and 271.4 F g^−1^ for CMC, CMC-Zn, CMC-K, and CMC-ZnK, respectively. At a higher current density of 10 A g^−1^, these values decreased to 160.0, 176.0, 198.0, and 244.0 F g^−1^, reflecting capacitance retention rates of 80.7%, 81.0%, 89.0%, and 90.0%, respectively. Among the materials, CMC-ZnK exhibited the most outstanding electrochemical performance, delivering a specific capacitance of 271.4 F g^−1^ at 1 A g^−1^ and retaining 244.0 F g^−1^ (90% retention) even under high-current operation at 10 A g^−1^. The performance enhancement stems from synergistic effects between zinc acetate and potassium citrate, coupled with N-O co-doping. Specifically, potassium citrate templates promote micropore formation while zinc acetate templates mediate mesoporous architecture, collectively establishing a hierarchical porous structure that shortens ion diffusion pathways and optimizes charge storage capacity. Additionally, the introduction of N-O co-doping generates abundant redox-active sites, further boosting electrochemical performance through enhanced surface reactivity. This dual strategy of structural engineering and heteroatom incorporation effectively addresses the common trade-off between capacitance and rate capability in carbon-based supercapacitors.

**Figure 8:**
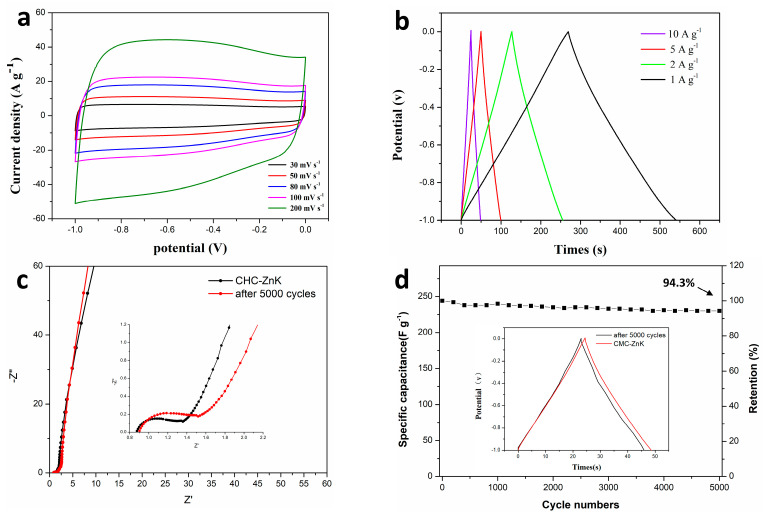
(**a**) CV curves of CMC-ZnK-800 at different scan rates; (**b**) GCD curves of CMC-ZnK at different current densities; (**c**) Nyquist plots of CMC-ZnK and 5000-cycle CMC-ZnK; (**d**) evaluation of specific capacitance versus number of cycles and comparison before and after 5000 cycles.

A comprehensive investigation of the electrochemical performance characteristics of the CMC-ZnK electrode was systematically conducted under a three-electrode configuration. Figure 8a presents the CV profiles of CMC-ZnK at varying scan rates (30 to 200 mV s^−1^), demonstrating well-maintained quasi-rectangular shapes even at the maximum rate of 200 mV s^−1^. The GCD curves (Figure 8b) exhibit symmetrical triangular performance under current densities varying from 1 to 10 A g^−1^, further confirming robust capacitive behavior. EIS measurements conducted in a three-electrode system with a 6 M KOH electrolyte (frequency range: 0.01 Hz–100 kHz) revealed critical charge transfer characteristics (Figure 8c). A semicircular arc is observed in the high-frequency region of the Nyquist plot, and a linear Warburg tail is observed in the low-frequency domain. The *x*-axis intercept is associated with the equivalent series resistance, while the semicircle diameter reflects charge transfer resistance, suggesting efficient ion diffusion kinetics [36]. Further analysis of the variations in the internal resistance of the material before and after the cycling process and electrochemical impedance analysis through equivalent circuit modeling revealed an exceptionally low equivalent series resistance (Rs) of 0.89 Ω for CMC-ZnK-800 (Figure S2). Remarkably, the material demonstrated outstanding resistance stability with negligible Rs variation after 5000 charge–discharge cycles. Long-term cyclic stability was evaluated by performing 5000 GCD cycles at 10 A g^−1^ in the voltage range of −1.0 to 0 V (Figure 8d). Remarkably, the material retained 94.3% of its initial capacitance, demonstrating superior electrochemical durability. This outstanding stability originates from the dual-template-derived porous structure and heteroatom doping, which synergistically mitigate structural degradation during repeated charge/discharge cycles. Moreover, the negligible increase in Rs throughout cycling demonstrates well-maintained structural integrity and interfacial stability between the electrode and electrolyte during extended operation.

**Figure 9:**
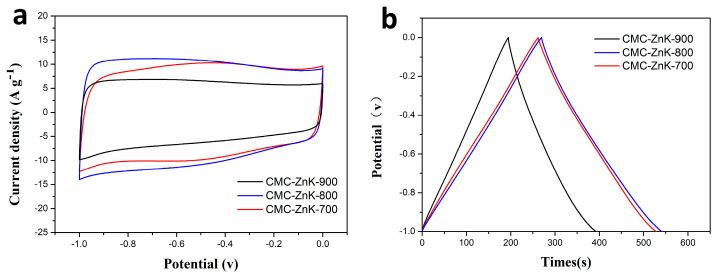
(**a**) CV curves of CMC-ZnK-700, CMC-ZnK-800, and CMC-ZnK-900 at 50mV∙s^−1^; (**b**) GCD curves of CMC-ZnK-700, CMC-ZnK-800, and CMC-ZnK-900 at 1 A g^−1^.

To systematically examine the influence regarding the effect of temperature on CMC-ZnK material synthesis, controlled experiments were conducted in a tube furnace under a N_2_ atmosphere at three distinct temperatures (700 °C, 800 °C, 900 °C), while maintaining identical precursor stoichiometry and activation duration. The corresponding samples were marked as CMC-ZnK-700, CMC-ZnK-800, and CMC-ZnK-900, respectively. As demonstrated in Figure 9a, all materials exhibit quasi-rectangular CV profiles at 50 mV s^−1^, along with symmetrical triangular GCD curves (Figure 9b), confirming dominant EDLC behavior. Rate capability analysis (Figure S3) revealed severe capacitive degradation in the sample carbonized at 900 °C, which exhibited a 60% reduction in specific capacitance at 10 A g^−1^ compared to the 800 °C sample. This performance decline is closely linked to critical structural changes, as discussed below. Elemental composition analysis (Table 2) demonstrates temperature-dependent nitrogen depletion, with N content decreasing from 10.57% (700 °C) to 4.11% (900 °C). The marked nitrogen loss at elevated temperatures likely originates from thermal volatilization of nitrogen/oxygen functional groups, significantly impairing electron transfer kinetics. Notably, the 800 °C carbonized CMC-ZnK exhibited optimal electrochemical performance, and the structural characteristics can be achieved through the following approaches: (1) preserved nitrogen/oxygen heteroatoms maintaining favorable conductivity, (2) developed hierarchical porosity enabling efficient ion transport, and (3) stable carbon matrix preventing structural collapse.

**Figure 10:**
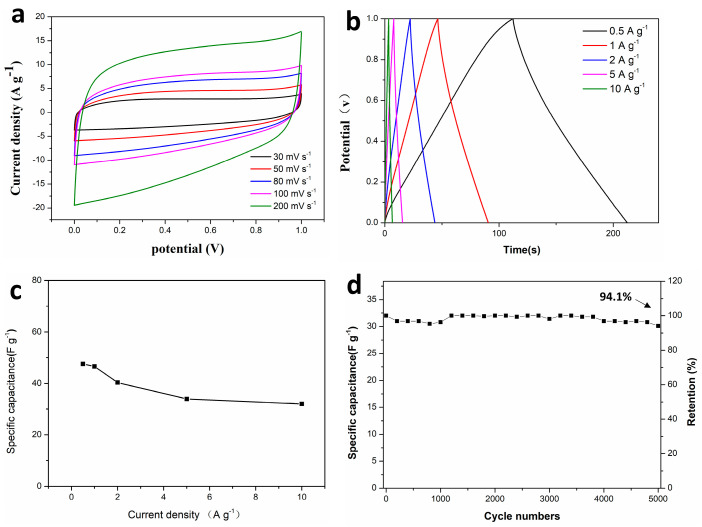

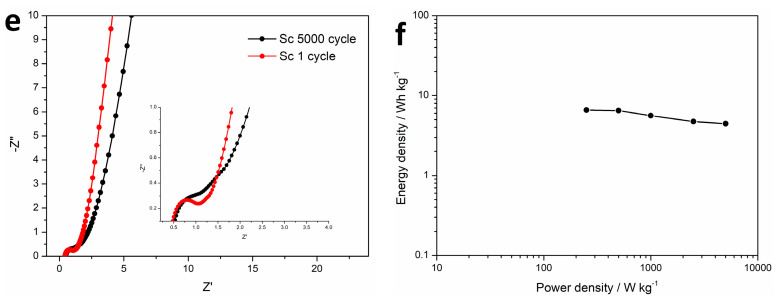
(**a**) CV curves of supercapacitor at different scan rates; (**b**,**c**) GCD curves of supercapacitor at different current densities and specific capacitances at different current densities; (**d**–**e**) cycling performance and coulombic efficiency at a current density of 10 A g^−1^ and Nyquist plots of supercapacitor before and after 5000 cycles; (**f**) Ragone plots of supercapacitor.

Electrochemical performance analysis of CMC-ZnK-800-based supercapacitors and the electrochemical performance of the CMC-ZnK-800 carbon material were systematically evaluated through symmetric supercapacitor assembly using a 3 M KOH aqueous electrolyte. Cyclic voltammograms recorded at scan rates spanning from 30 to 200 mV s^−1^ (Figure 10a) maintained quasi-rectangular profiles across all tested rates, demonstrating ideal electric double-layer capacitive characteristics with rapid charge propagation [37]. The preserved shape integrity at elevated scan rates confirms the enhanced ion diffusion kinetics and structural stability of the dual-doped carbon framework. The GCD curves exhibit nearly ideal isosceles triangular profiles at current densities ranging from 0.5 to 10 A g^−1^ (Figure 10b), demonstrating highly reversible charge storage behavior. The device delivered a specific capacitance of 46.6 F g^−1^ at 1 A g^−1^, while maintaining 68.7% capacitance retention (32 F g^−1^) even at an elevated current density of 10 A g^−1^ (Figure 10c). Long-term durability testing under aggressive conditions (10 A g^−1^ for 5000 cycles) demonstrated exceptional cycling stability with 94.1% capacitance retention (Figure 10d). Nyquist plot analysis (Figure 10e) revealed a moderate increase in internal resistance (Rs) post cycling, evidenced by semicircle radius expansion in the high-frequency region. This resistance elevation likely originates from partial structural degradation or electrolyte decomposition during prolonged operation. The Ragone plot (Figure 10f) highlights the device’s exceptional energy delivery characteristics, achieving an impressive energy density of 6.5 Wh kg^−1^ with a power density of 500 W kg^−1^.

## 4. Conclusions

This study successfully demonstrated the fabrication of a nitrogen–oxygen co-doped porous carbon material derived from chitosan and melamine, utilizing potassium citrate and zinc acetate as dual doping templates. The synergistic effects of this dual template significantly enhanced the material’s electrochemical properties compared to single-doped or non-doped counterparts. Notably, CMC-ZnK-800 material has a maximum specific surface area of 507.8 m^2^·g^−1^ compared to the comparison sample. The optimized nitrogen-to-oxygen ratio in CMC-ZnK-800 contributed to its superior electrochemical performance. The material delivered a specific capacitance of 271.4 F g^−1^ at 1 A g^−1^, with a remarkable retention of 90% (244 F g^−1^) even at 10 A g^−1^. However, increasing the carbonization temperature to 900 °C caused a drastic decline in performance, likely due to thermal degradation of functional groups containing nitrogen. We further tested the performance of CMC-ZnK-800 material as a symmetric supercapacitor electrode. The device achieves an energy density of 6.5 Wh kg^−1^ when operating at a power density of 500 W kg^−1^. It is anticipated that the present work will provide a useful compass to guide the future synthesis and preparation of doubly doped porous carbon materials based on chitosan and its derivatives.

## Figures and Tables

**Table 1:** C, N, and O element products of CMC, CMC-Zn, CMC-K, and CMC-ZnK.

Sample	C (at%)	N (at%)	O (at%)
CMC	79.26	12.18	8.55
CMC-Zn	70.87	12.02	17.11
CMC-K	77.1	6.09	16.82
CMC-ZnK	74.46	10.28	15.26

**Table 2:** C, N, and O element product of CMC-ZnK-700, CMC-ZnK-800, and CMC-ZnK-900.

Sample	C (at%)	N (at%)	O (at%)
CMC-ZnK-700	64.62	10.57	24.81
CMC-ZnK-800	74.46	10.28	15.26
CMC-ZnK-900	74.47	4.11	21.42

## Data Availability

The original contributions presented in this study are included in the article/Supplementary Materials. Further inquiries can be directed to the corresponding author.

## Supplementary Materials

The following supporting information can be downloaded at: https://www.mdpi.com/article/10.3390/polym17091198/s1, Figure S1. specific capacitances of CMC, CMC-Zn, CMC-K and CMC-ZnK at different current densities; Figure S2. CMC-ZnK-800 Simulation curve before and after fit; Figure S3. specific capacitances of CMC-ZnK-700, CMC-ZnK-800, and CMC-ZnK-900 at different current densities.
