# Supplementary material for: N- and O- Doped Porous Carbon Nanosheets Prepared from Templating Methodology for Supercapacitors
Source: Polymers (Basel). 2025 Apr 27;17(9):1198. doi: 10.3390/polym17091198 (PMC12073775; doi:10.3390/polym17091198)
Supplement: Supplementary file 1 [file polymers-17-01198-s001.zip › polymers-3587041-supplementary.pdf]

N- and O- Doped Porous Carbon Nanosheets Prepared from Templating Methodology for Supercapacitors

Baoning Zhu [1], Jinghua Liu [1,*], Qijun Zhong [1], Yaru Wen [1], Qianqian Dong [1], Yuhao Li [1], Qianqian Jin [1], Yao Lu [2,*]

[1] Liuzhou Key Laboratory of New Energy Vehicle Power Lithium Battery, Guangxi Engineering Research Center for Characteristic Metallic Powder Materials, School of Electronic Engineering, Guangxi University of Science and Technology, Liuzhou 545000, China; liujinghua@gxust.edu.cn (J.L.)
[2] Guangxi Key Laboratory of Green Processing of Sugar Resources, College of Biological and Chemical Engineering, Guangxi University of Science and Technology, Liuzhou 545006, China; luyao@163.com (J.C.);
* Correspondence: liujinghua@gxust.edu.cn (J.L.); luyan

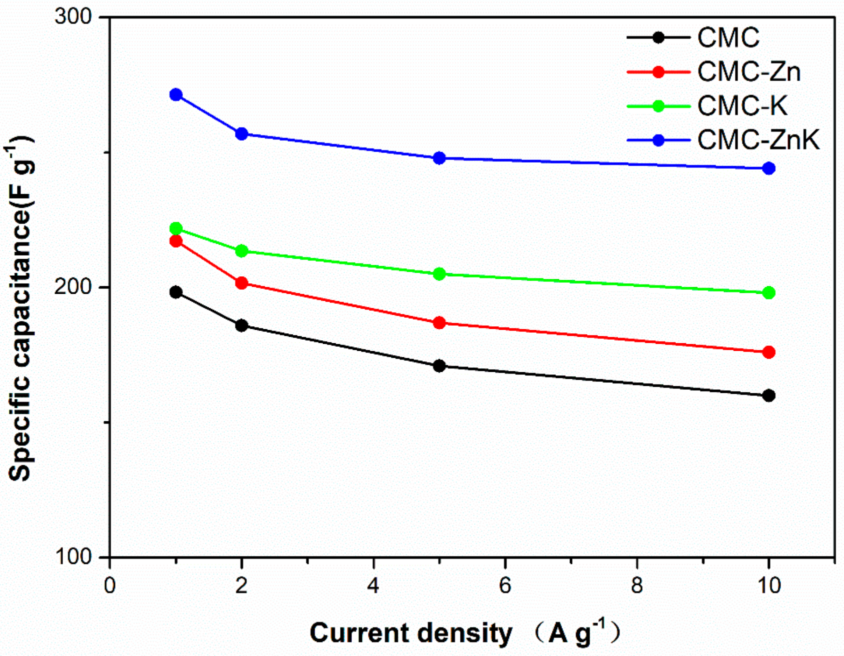

Figure S1. specific capacitances of CMC, CMC-Zn, CMC-K and CMC-ZnK at different current densities

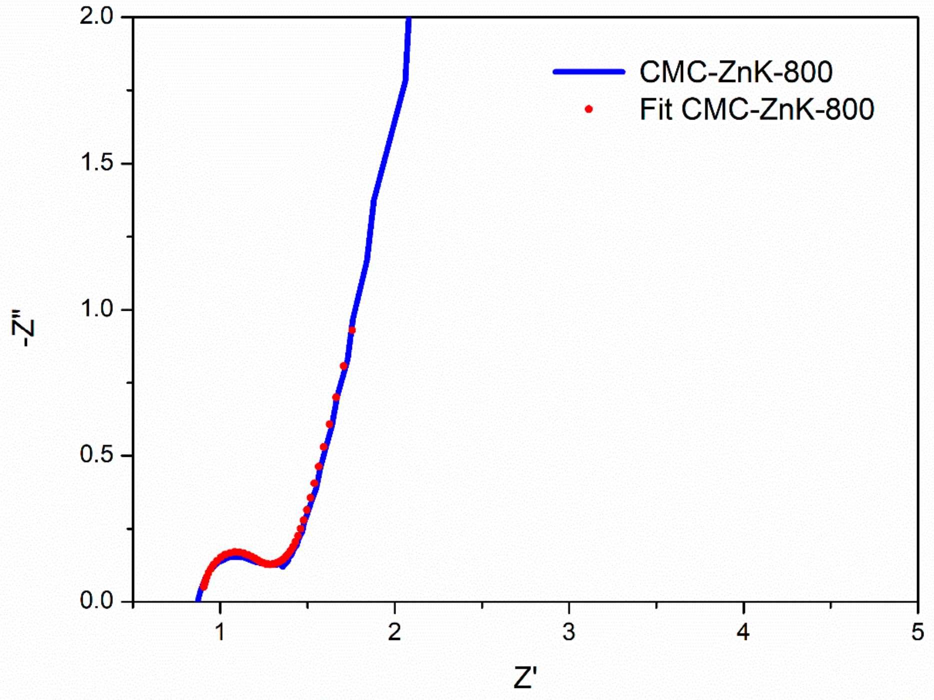

Figure S2. CMC-ZnK-800 Simulation curve before and after fit

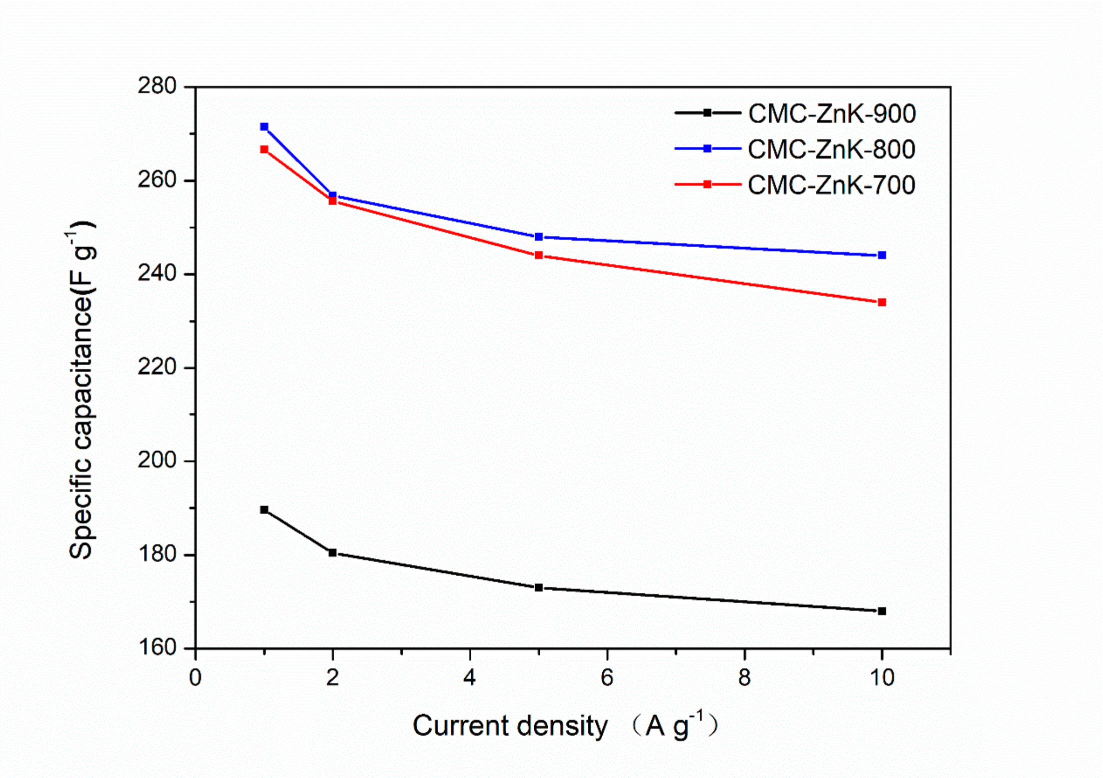

Figure S3. specific capacitances of CMC-ZnK-700, CMC-ZnK-800, and CMC-ZnK-900 at different current densities
